# Communicating Guideline Recommendations Using Graphic Narrative Versus Text-Based Broadcast Screensavers: Design and Implementation Study

**DOI:** 10.2196/27171

**Published:** 2021-12-13

**Authors:** Lauren Sinnenberg, Craig A Umscheid, Frances S Shofer, Damien Leri, Zachary F Meisel

**Affiliations:** 1 Department of Medicine Brigham and Women's Hospital Boston, MA United States; 2 Biological Sciences Division University of Chicago Chicago, IL United States; 3 Center for Healthcare Delivery Science and Innovation University of Chicago Medicine Chicago, IL United States; 4 Department of Emergency Medicine University of Pennsylvania Philadelphia, PA United States; 5 Center for Emergency Care Policy and Research University of Pennsylvania Philadelphia, PA United States; 6 Penn Medicine Center for Health Care Innovation Perelman School of Medicine University of Pennsylvania Philadelphia, PA United States

**Keywords:** medical informatics, screensaver, guideline dissemination, graphic narratives, health communication, workstation, clinical workstation, guidelines, medical education, education

## Abstract

**Background:**

The use of graphic narratives, defined as stories that use images for narration, is growing in health communication.

**Objective:**

The aim of this study was to describe the design and implementation of a graphic narrative screensaver (GNS) to communicate a guideline recommendation (ie, avoiding low-value acid suppressive therapy [AST] use in hospital inpatients) and examine the comparative effectiveness of the GNS versus a text-based screensaver (TBS) on clinical practice (ie, low-value AST prescriptions) and clinician recall.

**Methods:**

During a 2-year period, the GNS and the TBS were displayed on inpatient clinical workstations. The numbers of new AST prescriptions were examined in the four quarters before, the three quarters during, and the one quarter after screensavers were implemented. Additionally, an electronic survey was sent to resident physicians 1 year after the intervention to assess screensaver recall.

**Results:**

Designing an aesthetically engaging graphic that could be rapidly understood was critical in the development of the GNS. The odds of receiving an AST prescription on medicine and medicine subspecialty services after the screensavers were implemented were lower for all four quarters (ie, GNS and TBS broadcast together, only TBS broadcast, only GNS broadcast, and no AST screensavers broadcast) compared to the quarter prior to implementation (odds ratio [OR] 0.85, 95% CI 0.78-0.92; OR 0.89, 95% CI 0.82-0.97; OR 0.87, 95% CI 0.80-0.95; and OR 0.81, 95% CI 0.75-0.89, respectively; *P*<.001 for all comparisons). There were no statistically significant decreases for other high-volume services, such as the surgical services. These declines appear to have begun prior to screensaver implementation. When surveyed about the screensaver content 1 year later, resident physicians recalled both the GNS and TBS (43/70, 61%, vs 54/70, 77%; *P*=.07) and those who recalled the screensaver were more likely to recall the main message of the GNS compared to the TBS (30/43, 70%, vs 1/54, 2%; *P*<.001).

**Conclusions:**

It is feasible to use a graphic narrative embedded in a broadcast screensaver to communicate a guideline recommendation, but further study is needed to determine the impact of graphic narratives on clinical practice.

## Introduction

The use of graphic narratives is growing in health communication [1]. They are characterized as cohesive stories with an identifiable beginning, middle, and end that include characters, raise questions, provide resolution, and use images for narration. Graphic narratives have been successfully used by the American Cancer Society and the US Centers for Disease Control and Prevention in patient-facing communication [2,3]. The theoretical underpinnings for behavior change resulting from narratives include social cognitive theory and the theory of reasoned action [4].

Prior work in health communication has also evaluated the use of broadcast screensavers as educational tools for disseminating information to hospital staff, with mixed results [5-7]. No study to date has specifically examined the comparative effectiveness of different approaches to communicate messages using broadcast screensavers targeted to health care providers.

In this study, we describe the feasibility of designing and implementing a graphic narrative to communicate a guideline recommendation—namely, avoiding acid suppressive therapy (AST) in hospital inpatients at low risk of gastric stress ulcers—to health care providers through the use of broadcast screensavers, and we examine the comparative effectiveness of a graphic narrative screensaver (GBS) versus a text-based screensaver (TBS) on clinical practice and clinician recall [8-11].

## Methods

### Overview

This was a descriptive feasibility study as well as a quasi-experimental evaluative study that examined change in clinical practice over a 2-year period and included an experimental survey component to examine clinician recall. The study site was a single academic health care system consisting of three hospitals in an urban environment. The study was approved by the Institutional Review Board of the University of Pennsylvania.

### Graphic Narrative Design

The GNS and the TBS were designed to communicate the risk of unindicated AST prescription. We developed narratives through meetings in which feedback around the low-value prescription of AST was solicited in a semistructured manner from health care system faculty, nurses, fellows, and residents. The focus of these meetings was to elicit knowledge gaps, attitudes, and beliefs related to AST use. We contracted a graphic designer to create a slide that could be broadcast on the screensaver of inpatient clinical workstations and used established techniques and theoretical frameworks in narrative communication [4]. The slide was developed and refined in an iterative fashion in which the designer presented ideas and prototypes in three rounds to the research team, which was composed of decision scientists and clinicians. We simultaneously developed text-based, probabilistic descriptions of the published guidelines from the Choosing Wisely campaign to compare with the graphic narratives.

### Screensaver Intervention

Study screensavers were added to an existing, rotating deck of screensaver slides updated on the first of each month and displayed on all clinical workstation computers of all inpatient units in the three urban hospitals of our academic health care system. Slides were displayed from the deck in random order, lasting 18 seconds per slide. In most months, there are 10 or fewer slides in rotation, and rarely are there more than 20 slides in rotation. For the initial 3-month block of our study intervention period (October to December 2014), the GNS and TBS were both included in the slide deck for broadcasting. For the next 3 months (January to March 2015), only the TBS was included in the slide deck for broadcasting, followed by a 3-month block (April to June 2015) where only the GNS was included in the slide deck. The final 3 months of the study period (July to September 2015) included neither of the study screensavers.

### AST Prescriptions

New discharge prescriptions of AST for all low-risk inpatients were measured prior to, during, and following implementation of the intervention screensavers using data from our health care system’s electronic medical record (Allscripts). Patients were included if they were admitted and discharged during our study period of October 1, 2013, to September 30, 2015. Inpatients were defined as “low risk” using criteria from the American Society of Health-System Pharmacists guideline on AST use [12]. To ensure we included only low-risk inpatients in our analysis, the following patients were excluded: intensive care unit patients with an international normalized ratio of >1.9 or partial thromboplastin time of >54, patients on mechanical ventilation, patients with a history of or current peptic ulcer disease (ICD-9 [International Classification of Diseases, Ninth Revision] codes 531-533), and patients cared for on the clinical research unit, hospice, and gastroenterology medical or surgical service. Patients who were less than 18 years of age, left against medical advice, expired during hospitalization, or discharged to hospice were also excluded. AST was defined as any of the following: proton pump inhibitors (ie, lansoprazole, omeprazole, pantoprazole, dexlansoprazole, esomeprazole, and rabeprazole) or histamine H2-receptor antagonists (ie, cimetidine, famotidine, nizatidine, and ranitidine).

### Resident Physician Survey

One year after the screensavers were broadcast, an electronic questionnaire was emailed to all second- and third-year internal medicine resident physicians. First-year residents were excluded as they had not been exposed to the intervention.

To evaluate retention of guideline information, the survey had an experimental design. Participants were shown the slides from the screensavers with all written content deliberately blurred. Residents were then asked to recall if they had seen the slide and to describe the content of the slide from memory. Participants were also asked about prescribing patterns of AST, adverse effects of AST, and how their prescribing patterns had changed in the previous year. The survey was emailed four times to the study population. To compensate the residents for their participation, they were entered into a lottery for one of six US $25 gift cards or an Apple Watch.

### Statistical Analysis

Summary statistics, such as frequencies and percentages or means and SDs, were used to describe the patient population in terms of sex, race, age, hospital, and clinical service. Clinical discharge service was divided into six groups: (1) medicine (eg, general internal medicine), (2) surgery (eg, orthopedics, neurosurgery, general surgery, and urology), (3) medical subspecialty services (eg, oncology, cardiology, pulmonary, and infectious disease), (4) family medicine, (5) neurology, and (6) obstetrics and gynecology. The 2-year study period was divided into eight, 3-month blocks: prequarter 1, prequarter 2, prequarter 3, prequarter 4, GNS and TBS, TBS alone, GNS alone, and postquarter. To assess trends in AST prescriptions, logistic regressions that were modeled on receiving a new AST prescription on discharge, adjusted for sex, race, age, and hospital, were developed. The models for each 3-month period were compared to prequarter 4 (ie, the 3-month period prior to the intervention).

Standard summary statistics were used to describe participants in the survey. To compare differences in screensaver recall, chi-square and McNemar tests were used. All analyses were performed using SAS statistical software (version 9.4; SAS Institute).

## Results

### Graphic Narrative Design and Implementation

**Figure 1:**
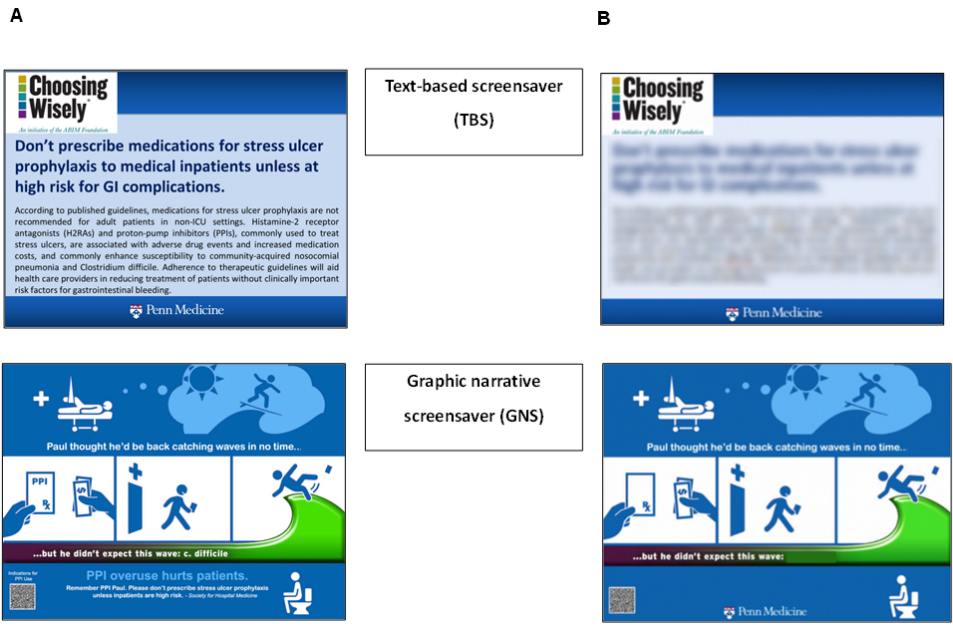
Screensaver interventions (A) and experimental survey design (B) containing screensavers blinded by blurring all content-specific text.

During our semistructured meetings, we found the following to be important features of graphic narrative design: crafting the guideline-based message into narrative form; creating a narrative that is attention grabbing, such that it attracts busy hospital staff; and ensuring that the graphic narrative is quickly comprehensible. We contracted a designer who was able to create a graphic narrative that met these key requirements (Figure 1).

### Effect of GNS and TBS on Acid Suppressive Therapy Prescriptions

During the 2-year period, 157,110 patients were admitted to one of the three study hospitals, of which 97,767 met the inclusion criteria (62.2%). The patient sample was 60.9% (n=59,495) male, 53.3% (n=51,017) White, and 41.1% (n=39,419) African American or Black, and had a mean age of 52.0 (SD 19.2) years. Most patients were discharged from surgical services (n=31,429, 32.1%), followed by obstetrics and gynecology (n=21,117, 21.6%), internal medicine (n=20,934, 21.4%), internal medicine subspecialty (n=20,592, 21.1%), neurology (n=2526, 2.6%), and family medicine (n=1169, 1.2%) services. A total of 56.0% (n=54,799) of the patients were discharged directly home.

**Figure 2:**
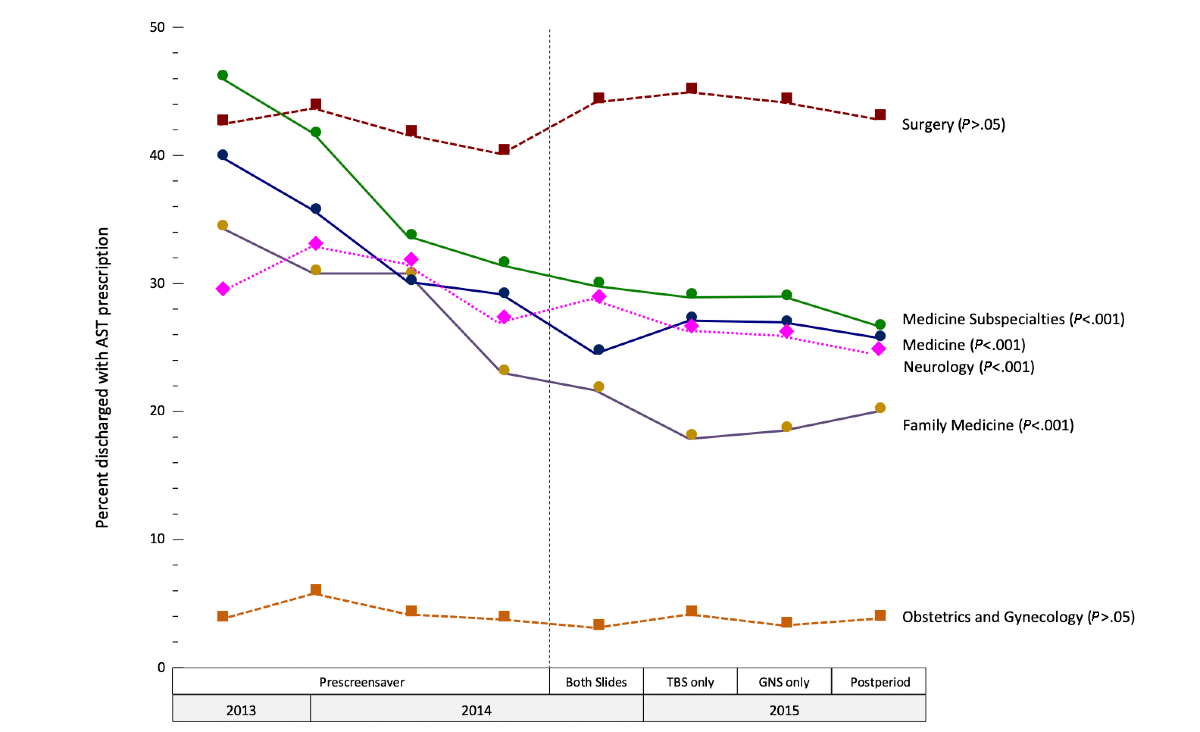
Acid suppressive therapy (AST) prescription patterns during the study period. GNS: graphic narrative screensaver; TBS: text-based screensaver.

After adjusting for sex, race, age, and hospital, for both medicine and medicine subspecialty services combined, the odds of receiving an AST prescription after the screensaver interventions were implemented was lower for all four quarters (ie, GNS and TBS, TBS alone, GNS alone, and postquarter) compared to prequarter 4 (odds ratio [OR] 0.85, 95% CI 0.78-0.92; OR 0.89, 95% CI 0.82-0.97; OR 0.87, 95% CI 0.80-0.95; and OR 0.81, 95% CI 0.75-0.89, respectively; *P*<.001 for all comparisons). There were no statistically significant decreases for the other services. These declines appear to have begun prior to screensaver implementation (Figure 2).

### Resident Physician Survey

Of the 97 residents invited to participate, 70 (72%) completed the survey. The median age of participants was 29 (IQR 2) years, and 51% (n=36) were male. Most residents indicated that they could recall seeing both the GNS and TBS (n=43, 61%, vs n=54, 77%; *P*=.07). When those who recalled seeing the screensavers were asked where they had seen the image, 93% (40/43) recalled that the GNS was a screensaver, compared to 24% (13/54) for the TBS. Furthermore, 70% (30/43) could recall the main topic of the GNS, compared to 2% (1/54) of the TBS (*P*<.001). Many residents indicated that they prescribed fewer ASTs than they did 1 year prior (38/70, 54%), and 8% (3/38) of these participants directly attributed their change to the screensavers.

## Discussion

We sought to design a GNS that communicated guideline recommendations. In a design process that included semistructured meetings with key stakeholders as well as the efforts of a professional graphic designer, we found that it was feasible to create and disseminate a graphic narrative to summarize and communicate guideline recommendations.

In our study period, approximately one-quarter of patients were discharged with inappropriate AST prescriptions, but these AST prescriptions decreased over time on the nonsurgical services. This decrease, however, appeared to have begun prior to the screensaver intervention. It is possible that there were ongoing efforts on the nonsurgical services to reduce unnecessary AST prescriptions, and it is unknown whether the screensaver initiative may have potentiated this effect. The intervention seems to have had a lower effect on the surgical services, potentially related to less time spent by surgical service residents on the computer workstation.

Our study raises the possibility that GNSs may be useful tools for disseminating guideline recommendations. It is possible that the residents recognized the Choosing Wisely logo in the TBS leading to improved recognition compared to the GNS, although this difference was not statistically significant. This did not result in improved content-specific recall, however, which was significantly greater for the GNS compared to the TBS. This is consistent with prior work that demonstrated improved information delivery to clinicians when content was presented in narrative form as opposed to a summary statement form [13]. Further work should be done to understand whether graphic narratives have an impact on clinical practice and, if so, what features of graphic narratives improve information delivery.

Our study has limitations. We incorporated our screensavers into an existing broadcast screensaver program within an academic health care system. Guideline dissemination via broadcast screensavers may prove more challenging in nonacademic settings without established broadcast screensaver programs. In addition, our quasi-experimental design prevents us from isolating the effects of our intervention from other interventions that may have concurrently affected AST prescription rates. The survey portion of our work had a relatively small sample size, which may have limited our ability to detect differences in recognition and recall. Future work should employ a randomized design to best isolate the effect of GNSs from other interventions.

In conclusion, it is feasible to use a graphic narrative embedded in a broadcast screensaver to communicate a guideline recommendation, but further study is needed to determine the impact of graphic narratives on clinical practice.

## Abbreviations

AST: acid suppressive therapy
GNS: graphic narrative screensaver
ICD-9: International Classification of Diseases, Ninth Revision
TBS: text-based screensaver
